# The Activation of Nrf2 and Its Downstream Regulated Genes Mediates the Antioxidative Activities of Xueshuan Xinmaining Tablet in Human Umbilical Vein Endothelial Cells

**DOI:** 10.1155/2015/187265

**Published:** 2015-11-22

**Authors:** Lingxin Xiong, Jingshu Xie, Chenxue Song, Jinping Liu, Jingtong Zheng, Chuangui Liu, Xiaotian Zhang, Pingya Li, Fang Wang

**Affiliations:** ^1^Department of Pathogeny Biology, Basic Medical College, Jilin University, Changchun 130021, China; ^2^Department of Pharmaceutical Sciences, Jilin University, Changchun 130021, China

## Abstract

Epidemiological studies have verified the critical role that antioxidative stress plays in protecting vascular endothelial cells. The aims of the present study were to investigate the antioxidative activities and differential regulation of nuclear erythroid-related factor 2- (Nrf2-) mediated gene expression by Xueshuan Xinmaining Tablet (XXT), a traditional Chinese medicine with the effect of treating cardiovascular diseases. The antioxidative activities of XXT were investigated using quantitative real-time PCR (qPCR), a PCR array, and western blotting. Our results indicated that XXT exhibited potent antioxidative activities by suppressing the levels of hydrogen peroxide- (H_2_O_2_-) induced reactive oxygen species (ROS) in human umbilical vein endothelial cells (HUVECs). We were also conscious of strong Nrf2-mediated antioxidant induction. XXT enhanced the expressions of Keap1, Nrf2, and Nrf2-mediated genes, such as glutamate-cysteine ligase modifier subunit (GCLM), NAD(P)H: quinine oxidoreductase 1 (NQO1), heme oxygenase 1 (HMOX1), and glutathione peroxidase (GPX) in HUVECs. In summary, XXT strongly activated Nrf2 and its downstream regulated genes, which may contribute to the antioxidative and vascular endothelial cell protective activities of XXT.

## 1. Introduction

Traditional Chinese medicine (TCM), guided by the theory of traditional Chinese medical science, has been used for over 5,000 years mainly in China and other Asian countries. Xueshuan Xinmaining Tablet (XXT) formula is composed of ten traditional Chinese medicines:* Chuanxiong Rhizoma, Salviae Miltiorrhizae Radix et Rhizoma, Hirudo, Bovis Calculus, Moschus, Pubescent Holly Root, Sophorae Flos, total ginsenoside of ginseng stems and leaves, Borneolum syntheticum*, and* Bufonis venenum*. Previously, we identified the components of XXT with a high performance liquid chromatography system and obtained the fingerprint of the XXT extract [1]. XXT has been widely used in the cerebral thrombosis and coronary heart disease in China for activating blood circulation and removing blood stasis for more than a decade [2, 3]. Oxidative stress is associated with all cardiovascular diseases, including hypertension and hypercholesterolaemia, and is involved in the formation of these diseases through the activation of NADPH oxidases that generate reactive oxygen species (ROS) [4]. Thus, we investigated the antioxidation of XXT* in vitro*.

The Nrf2 system is considered to be a major cellular defense mechanism against oxidative stress. Nrf2 plays an important role in cellular defense and in improving the removal of ROS by activating genes that encode phase II detoxifying enzymes and antioxidant enzymes, such as GCLM, NQO1, HMOX1, GPX, and glutathione S-transferases (GST) [5]. Under basal conditions, Nrf2 appears to be associated with actin-binding Keap1, which forms the Keap1-Nrf2 complex. The Keap1-Nrf2 complex prevents Nrf2 from entering the nucleolus, which promotes its proteasomal degradation. Upon treatment in the cells with oxidants including H_2_O_2_, oxidative stress and conformational changes occur as a result of the oxidation of thiol-sensitive amino acids that are present in the Keap1-Nrf2 complex and may drive the dissociation of Nrf2 from Keap1. Nrf2 then translocates into the nucleus, where it binds to the antioxidant response element (ARE) of ARE target genes, which leads to enhanced antioxidant enzyme expression [6–8].

In the present study, we aimed to investigate the transcriptional regulation of Nrf2 and its downstream regulated genes, including GCLM, NQO1, HMOX1, and GPX, which controlled the expression of antioxidative genes and ROS in order to examine whether XXT had antioxidative activities that were mediated by the regulation of Nrf2.

## 2. Materials and Methods

### 2.1. Materials

Xueshuan Xinmaining Tablet (XXT) was provided by Jilin Huakang Pharmaceutical Co., Ltd. Human umbilical vein endothelial cells (HUVECs) were purchased from American Type Culture Collection (Manassas, VA, USA). TACS MTT Cell Proliferation Assay Kit and GPX enzyme-linked immunosorbent assay (ELISA) were purchased from R&D Systems (Minneapolis, MN, USA). Reactive Oxygen Species Assay Kit was obtained from Beyotime (Shanghai, China). Human Oxidative Stress PCR Array was purchased from Qiagen (Valencia, CA, USA). Anti-Nrf2, Keap1, GCLM, NQO1, HMOX1, and anti-Keap1 antibodies were purchased from Santa Cruz Biotechnology (Santa Cruz, CA, USA). Secondary antibodies were obtained from Sigma-Aldrich (St. Louis, MO, USA).

### 2.2. Cell Culture and Treatment

The HUVECs were cultured in Iscove's Modified Dulbecco's Medium (IMDM) (Invitrogen, Carlsbad, CA, USA) that was supplemented with 15% fetal bovine serum (FBS) (Hyclone, Logan, UT, USA) in a 37°C incubator in the presence of 5% CO_2_. The media were replaced with IMDM containing 2% FBS during the test period. The cells were divided into five groups: a control group (NC), an oxidative stress model group (Model), and three XXT-treated groups, including a high dose (200 *μ*g/mL), a middle dose (100 *μ*g/mL), and a low dose group (50 *μ*g/mL). The cells in the NC group were cultured in IMDM containing 2% FBS. The cells in the Model group were treated with 0.2 mM H_2_O_2_ for 4 h. The cells in the XXT-treated groups were pretreated with XXT (200, 100, and 50 *μ*g/mL) for 24 h prior to the treatment with H_2_O_2_ (Beijing, China) [9]. The dose of H_2_O_2_ was based on IC_50_ of H_2_O_2_, while the dose of XXT was based on the appropriate concentration of XXT, which was determined by a screening study. Cell viability was assessed via the MTT method using the TACS MTT Cell Proliferation Assay Kit.

### 2.3. Measurement of Intracellular ROS Levels

The intracellular ROS levels of the HUVECs were detected using the Reactive Oxygen Species Assay Kit. To measure the levels of ROS, the HUVECs were incubated with 10 *μ*M 2′,7′-dichlorodihydrofluorescein diacetate (DCFH-DA) at 37°C in the dark for 20 min after treatment. The cells were then assayed using flow cytometry.

### 2.4. Oxidative Stress PCR Array

The Human Oxidative Stress PCR Array was used to evaluate the relative expression of 84 oxidative stress-related genes. Total RNA was isolated from the HUVECs using the RNeasy Mini Kit (Qiagen, Valencia, CA). UNIC 2800 UV/VIS Spectrophotometer was used to assess the quantity and quality of the RNA extracts by measuring the absorbance at 260 and 280 nm. Total RNA was purified using RNase-Free DNase Set (Qiagen, Valencia, CA). cDNA was generated by reverse transcription of 20 ng of total RNA from each sample using the RT^2^ First Strand Kit (Qiagen, Valencia, CA) and was then combined with the RT^2^ SYBR Green ROX qPCR Mastermix in 96-well arrays. Thermal cycling was performed using an ABI Prism SDS 7300 system (Applied Biosystems). Gene expression was compared using C_t_ values and the results were calculated using ^ΔΔ^C_t_ method with normalization to the average expression levels of five common genes (ACTB, B2M, GAPDH, HPRT, and RPL13A) [10].

### 2.5. Quantitative Real-Time PCR Assays

Total RNA was isolated from the HUVECs using the RNeasy Mini Kit (Qiagen, Valencia, CA) and the quantity was measured using a spectrophotometer, as previously described. Total RNA was transcribed using the PrimeScript RT Reagent Kit with the gDNA Eraser (TaKaRa, Dalian, China). The primers that were used for qPCR are listed in Table 1 (Sangon Biotech, Shanghai, China). The cDNA was assessed using Real-Time PCR with SYBR Premix Ex Taq (TaKaRa, Dalian, China). Thermal cycling was performed using an ABI Prism SDS 7300 system (Applied Biosystems). Gene expression was compared according to C_t_ values, as previously described.

### 2.6. Western Blotting

After treatment, the cells were collected and lysed in RIPA buffer (Sigma, St. Louis, MO). 25 *μ*g protein per lane was loaded onto SDS-PAGE gels and then transferred onto nitrocellulose membranes (Millipore Corp., Billerica, MA, USA). The membranes were then blocked and probed using antibodies that were raised against the target proteins, including *β*-actin, Nrf2, Keap1, GCLM, NQO1, and HMOX1, and were incubated with appropriate secondary antibodies for 2 h. A coloration solution mixture (Beyotime, Jiangsu, China) was added and the immunoreactive bands were determined after exposure to the solution.

### 2.7. Statistical Analyses

The data were presented as mean values ± SD. Statistical analyses were performed using Student's *t*-test. *P* < 0.05 was considered significant. Statistical comparisons among three groups were performed using one-way ANOVA. The qPCR results were analyzed using the nonparametric Mann-Whitney *U* statistical test. These analyses were performed using SPSS software (version 18, USA) [11, 12].

## 3. Results 

### 3.1. H_2_O_2_-Induced Cytotoxicity in a Dose-Dependent Manner

**Figure 1:**
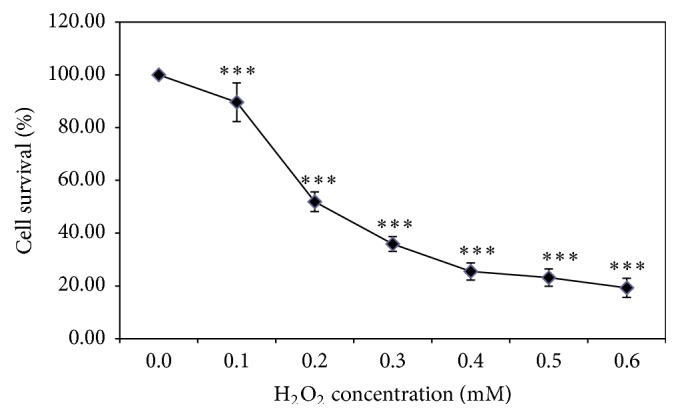
Effects of H_2_O_2_ treatment on the viability of HUVECs.

Cells were exposed to different concentrations of H_2_O_2_ for 4 h to examine H_2_O_2_-induced oxidative stress in HUVECs. The results revealed that H_2_O_2_ exposure led to oxidative stress in a concentration-dependent manner. There was 51.74% (*P* < 0.001) reduction in cell number when the cells were treated with 0.2 mM H_2_O_2_ (Figure 1). Therefore, this concentration was taken to be IC_50_ of H_2_O_2_ in HUVECs.

The cells were exposed to 0–0.6 mM H_2_O_2_ for 4 h and cell viability was evaluated using the TACS MTT Cell Proliferation Assay Kit. The percentage of cell survival was determined using the ratio of the optical density (OD) of the test sample to the OD of the control × 100%. The data shown are the means ± SD of three separate experiments in six replicate wells, ^*∗∗∗*^ *P* < 0.001 compared to the control.

### 3.2. Effects of XXT on H_2_O_2_-Induced Oxidative Stress in HUVECs

**Figure 2:**
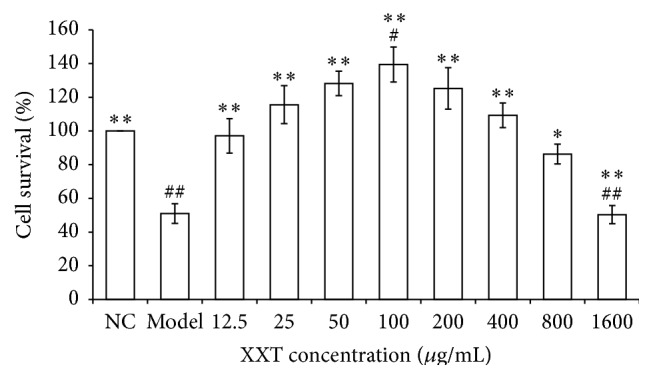
Effects of XXT on H_2_O_2_-induced oxidative stress in HUVECs.

Incubation of HUVECs with H_2_O_2_ decreased cell viability significantly. This decrease in viability was inhibited by XXT (Figure 2). The results indicated that XXT at the concentrations in the range of 25 to 400 *μ*g/mL promoted cell proliferation and prevented H_2_O_2_-induced reductions in HUVEC viability as compared to the Model group. However, XXT at excessive concentrations induced cellular injury.

Cell viability was evaluated using the MTT assay. NC: normal control cells were cultured in IMDM containing 2% FBS. Model: oxidative stress model group cells were treated with 0.2 mM H_2_O_2_ for 4 h. The remaining groups of cells were pretreated for 24 h with XXT (12.5, 25, 50, 100, 200, 400, 800, and 1600 *μ*g/mL) prior to treatment with H_2_O_2_. The percentage of cell survival was determined by the ratio of the optical density (OD) of the test samples to the OD of the control × 100%. The data are presented as the means ± SD of measurements that were performed in triplicate in six replicate wells, ^*∗*^ *P* < 0.05 and ^*∗∗*^ *P* < 0.01 versus the model.

### 3.3. Effects of XXT on ROS Production

**Figure 3:**
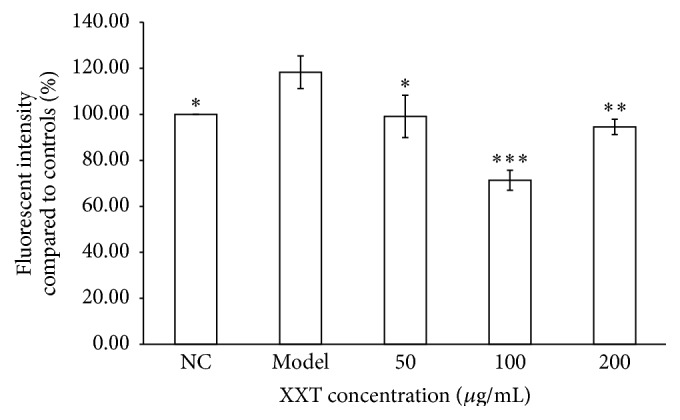
Effects of XXT on intracellular ROS in HUVECs.

The formation of ROS is indicative of oxidative stress. There were significantly higher ROS levels to 118.26% in H_2_O_2_-treated HUVECs compared to normal control cells (Figure 3). After XXT pretreatment, the level of ROS decreased significantly, indicating that XXT played an important role in reducing H_2_O_2_-induced oxidative stress in HUVECs.

Intracellular ROS, as measured using FACS Calibur flow cytometry (BD Biosciences, Africa), in HUVECs are shown after treatment with H_2_O_2_ alone and pretreatment with XXT. NC: normal control cells were cultured in IMDM containing 2% FBS. Model: oxidative stress model group cells were treated with H_2_O_2_ (0.2 mmol/L) for 4 h. The remaining groups of cells were pretreated for 24 h with XXT (50, 100, and 200 *μ*g/mL) prior to being treated with H_2_O_2_. The data are presented as the means ± SD of measurements that were performed in triplicate, ^*∗*^ *P* < 0.05 and ^*∗∗*^ *P* < 0.01 versus the Model group.

### 3.4. XXT Induced the Differential Regulation of Oxidative Stress-Related Genes

We examined the expressions of 84 oxidative stress-related genes in the cells from the Model group and 100 *μ*g/mL XXT-treated group using the Human Oxidative Stress PCR Array. Twenty genes were significantly upregulated (Fold Change > 2) and 18 genes were downregulated (Fold Change < 0.5) following XXT treatment. Among them, 8 genes were significantly upregulated and 9 genes were significantly downregulated. We chose the oxidative stress-related genes by screening for credible traffic between three specimen replication experiments (Table 2).

### 3.5. Effects of XXT on Nrf2 mRNA Expression Levels and Its Transactivated Target Gene Expression in HUVECs

**Figure 4:**
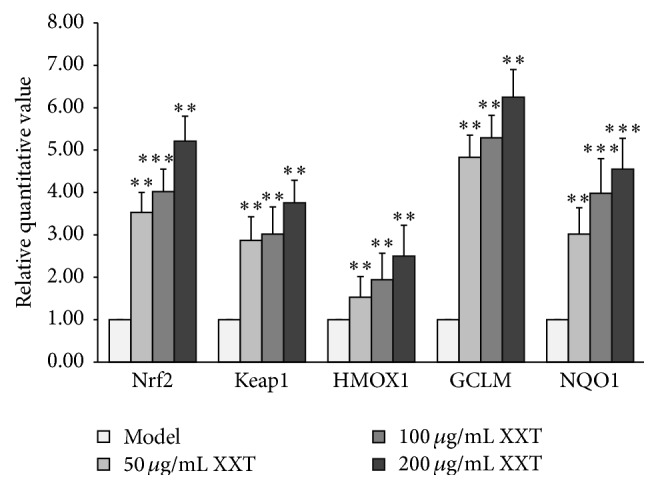
Effects of XXT on Nrf2 and Keap1 mRNA expression levels in HUVECs.

To investigate whether XXT affected the transcriptional regulation of Nrf2 and its transactivated target genes, qPCR analyses were performed. XXT at the concentrations of 50, 100, and 200 *μ*M induced the Keap1 activity to 3.53, 4.02, and 5.21, respectively, when compared to the Model group (Figure 4). Nrf2 expression was also induced by XXT at the concentrations of 50, 100, and 200 *μ*M to 2.87, 3.2, and 3.76, respectively, as compared to the Model group (Figure 4). The expressions of the transcriptional regulation genes HMOX1, GCLM, and NQO1, which are members of the Nrf2 downstream genes, were also significantly increased by XXT treatment (Figure 4).

NC: normal control cells were cultured in IMDM containing 2% FBS. Model: oxidative stress model group cells were treated with H_2_O_2_ (0.2 mM) for 4 h. The remaining groups of cells were pretreated with 50, 100, and 200 *μ*g/mL of XXT for 24 h prior to being treated with H_2_O_2_. After RNA extraction, 1 *μ*g of total RNA for each sample was converted to cDNA and analyzed using quantitative real-time RT-PCR. The differences in C_t_ values of Keap1, Nrf2, HMOX1, GCLM, NQO1, and *β*-actin gene were calculated to determine the levels of expression of Keap1 and Nrf2. The experiments were conducted in triplicate. The data are presented as the means ± SD, ^*∗*^ *P* < 0.05, ^*∗∗*^ *P* < 0.01, and ^*∗∗∗*^ *P* < 0.001 compared to the Model group.

### 3.6. Effects of XXT on the Protein Expression Levels of Nrf2 and Its Transactivated Target Genes in HUVECs

**Figure 5:**
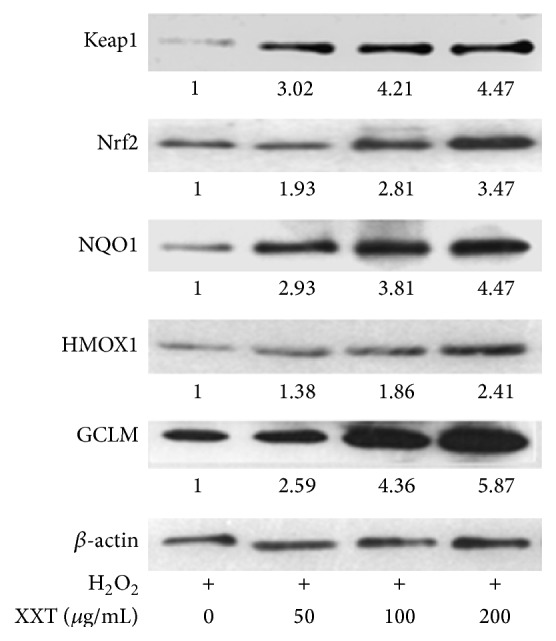
Effects of XXT on the protein expression levels of Keap1, Nrf2, HMOX1, GCLM, and NQO1 in HUVECs.

To investigate whether XXT-mediated antioxidant gene activation was controlled by the Nrf2 signaling pathway, the expression levels of these genes were measured using western blot analyses.  Figure 5 showed the levels of Keap1, Nrf2, HMOX1, GCLM, and NQO1. These genes were associated with oxidative stress, and the expressions of these proteins in the H_2_O_2_-exposed HUVECs were therefore examined. The results showed that their expressions were upregulated in this study. XXT treatment significantly induced the expressions of Keap1, Nrf2, HMOX1, GCLM, and NQO1 in HUVECs when compared to the Model group [13, 14].

HUVECs were cultured in IMDM containing 2% FBS. The second group of cells was incubated with 0.2 mM of H_2_O_2_ for 4 h. The remaining three groups of cells were pretreated with 50, 100, and 200 *μ*g/mL of XXT for 24 h prior to being incubated with H_2_O_2_. The proteins were extracted from the cell lysates after treatment. Western blot analyses were performed using Keap1, Nrf2, HMOX1, GCLM, and NQO1 antibodies. *β*-actin served as the housekeeping control.

Under basal conditions, newly synthesized Nrf2 is constitutively bound to Keap1 in the cytoplasm and forms a dimer, Nrf2-Keap1. Keap1 is a cytosolic protein that inhibits Nrf2 signaling by promoting the degradation of Nrf2 through the proteasomal pathway. When oxidants, such as ROS, react with the redox reactive cysteines in Keap1, Nrf2 is released from Keap1, allowing the transcription factor Nrf2 to translocate into the nucleus. In the nucleus, Nrf2 dimerizes with Maf proteins and binds to the ARE, which is located in the promoter region of phase II and antioxidative genes, and triggers the transcription of ARE-regulated genes. The critical role of Nrf2 in protecting cells from oxidative stress is performed by enhancing the expression of detoxifying metabolizing enzymes and maintaining oxidative stress homeostasis by producing antioxidant enzymes. With treatment by tetramethylpyrazine, XXT exerts strong antioxidative properties and protects vascular endothelial cells by suppressing the levels of ROS and enhancing the activity of Keap1, Nrf2, and Nrf2-mediated genes.

## 4. Discussion

Reactive oxygen species (ROS) has been recognized in playing a role in normal intracellular signaling that is required for survival and has also been shown to contribute to cytotoxicity [15]. Oxidative stress is defined as an imbalance between ROS production and the endogenous antioxidant mechanisms that counteract the effects of ROS and repair the resulting damage [16]. ROS leads to widespread and indiscriminate cellular damage, which is caused by either reduced detoxification or increased ROS levels [17]. H_2_O_2_ is considered to be one of the main ROS types and to rapidly cross cell membranes and generate different types of ROS, such as hydroxyl radicals, which are highly harmful [18]. Therefore, we evaluated the efficacy of XXT against H_2_O_2_-induced oxidative stress and ROS generation in HUVECs. Our results indicated that 0.2 mM H_2_O_2_ efficiently stimulated the release of ROS, thus leading to oxidative stress in HUVECs (Figures 1 and 3).

XXT is used for heart protection and treatment for cerebral vessel diseases in China. In agreement with the results of previously published studies, our data indicated that XXT strongly inhibited thrombosis and thereby protected vascular endothelial cells and maintained antioxidative properties. Our previous results revealed that XXT improved hemorheology and prevented thrombosis* in vivo* and acute blood stasis in rats and increased the levels of nitric oxide (NO) and nitric oxide synthase (NOS) in plasma. The mechanisms of XXT's actions may involve the unblocking of the G0-G1 period of the cell cycle, which is affected by H_2_O_2_. However, the mechanisms of action and the precise targets of XXT have not yet been definitively elucidated and need further studies. This study indicated that drug concentrations in the range of 25–400 *μ*g/mL promoted cell proliferation but did not cause oxidative damage or cell death when the fluctuation of concentrations was larger than this range (Figure 2). Therefore, we chose XXT at 50, 100, and 200 *μ*g/mL to perform the following tests. Then, we evaluated the efficacy of XXT against H_2_O_2_-induced oxidative stress and ROS levels in HUVECs. Pretreatment of the cells with XXT (50, 100, and 200 *μ*g/mL) greatly improved the cell survival rate and attenuated H_2_O_2_-induced ROS production (Figure 3). Thus, the results of our study demonstrated that XXT possessed significant ROS scavenging and protective activities in HUVECs. Therefore, the reliable therapeutic efficacy of XXT may correlate with its antioxidation activities, although the precise targets or pathways by which XXT intervenes in these processes require further studies.

PCR array analyses of oxidative stress-related genes were used to determine the mechanisms of XXT's actions. The results revealed that 8 genes were upregulated and 9 genes were downregulated in a statistically significant manner (Table 2). Among them, we found that GCLM, NQO1, HMOX1, and GPX6 gene expressions were mediated by the transcription factor Nrf2. Cells contain highly regulated defense systems, including the redox-sensitive Nrf2 pathway [19, 20]. Accumulating lines of evidence indicate that many drugs may protect cells and the cardiovascular system from oxidative damage by activating the Nrf2 signaling pathway, which leads to the upregulation of antioxidant genes [21–23]. In the present study, we thus focused on the antioxidant effects of XXT.

**Figure 6:**
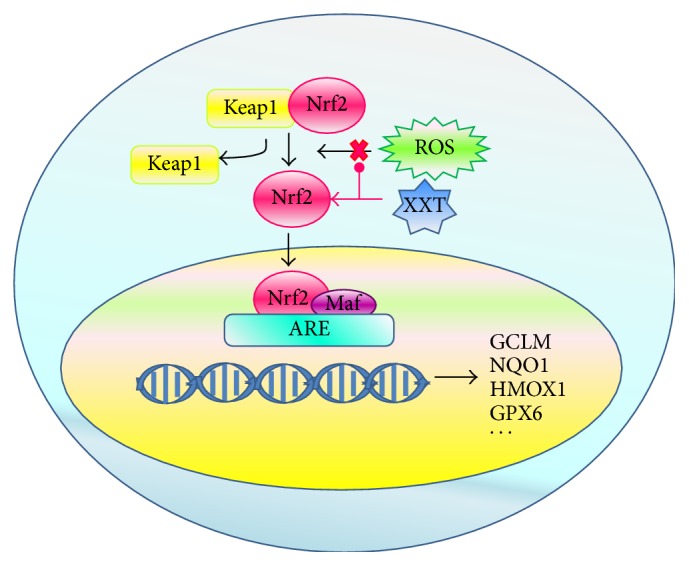
Schematic representation of XXT activities on Keap1-Nrf2-ARE pathway.

ROS levels influence the expressions of a number of genes that are involved in the regulation of the redox state through a negative feedback loop. One of the most extensively studied mediators of this gene expression regulatory system is the transcription factor Nrf2 [24]. Under physiological conditions, Nrf2 interacts with its inhibitor Keap1, a protein that contains several reactive cysteine residues [25]. When exposed to various stimuli, such as oxidative stress and certain antioxidants, the Keap1-Nrf2 complex is disrupted by modifying two cysteine residues of Keap1 [26], which allows the cytoplasmic-to-nuclear translocation of Nrf2. In the nucleus, Nrf2 dimerizes with small Maf proteins and binds to the ARE, which increases the gene expressions of phase II detoxifying and/or antioxidant enzymes, such as GST, SOD, CAT, GPX, NQO1, and HMOX1 [27, 28] (Figure 6). In the present study, XXT suppressed the battery of oxidative stress-induced activities by increasing the expressions of Nrf2 and its downstream active genes. Our results indicated that the mRNA transcriptions and expressions of Keap1, Nrf2, and Nrf2 downstream target genes, such as HMOX1, NQO1, GCLM, and GPX, were significantly induced by XXT treatment as compared to those in the Model cells (Figures 4 and 5). Furthermore, XXT reduced the mRNA expressions of the Nrf2-mediated genes CAT and GST, which possessed strong antioxidant and free radical inhibitory effects (Table 2). We hypothesized that other signaling pathways might participate in the regulatory effects of these two cytokines. These data suggested that XXT blocked the early stages of H_2_O_2_-induced activation of the Keap1-Nrf2-ARE pathway, inhibited the subsequent activity of the downstream pathway, and restored the balance of the Keap1-Nrf2-ARE pathway. The excess accumulation of Nrf2 in the cells is considered to be a factor indicating that oxidative damage has occurred and eventually leads to cellular protection against oxidative stress [29, 30]. Based on the mRNA and protein expression levels of Keap1 and Nrf2, as well as the Nrf2-mediated transcriptional regulation of GCLM, NQO1, HMOX1, and GPX, XXT was able to inhibit ROS levels and promote the accumulation of Nrf2, thereby protecting the HUVECs. The expression of Nrf2-ARE pathway is dependent upon modification of the Keap1-Nrf2 complex and Nrf2 nuclear translocation, and several signaling pathways are associated with these processes. Taken together, the regulation of the downstream kinases involved is a valuable tool for the investigation of Keap1-Nrf2 complex-controlled gene transcription [31].

## 5. Conclusion

In summary, our study indicates that the Nrf2 and its downstream genes play an important role in the regulation of antioxidative pathways. Our findings reveal that the XXT exerts strong antioxidative properties and protects vascular endothelial cells. Thus, the antioxidative abilities of XXT may potentially be used for the prevention of EC damage.

## Figures and Tables

**Table 1:** Primers used for quantitative real-time PCR.

Gene	Forward	Reverse
HuActin	5′-CTGGAACGGTGAAGGTGACA-3′	5′-AAGGGACTTCCTGTAACAATGCA-3′
Nrf2	5′-CAGCGACGGAAAGAGTATGA-3′	5′-TGGGCAACCTGGGAGTAG-3′
Keap1	5′-GGCTGTCCTCAATCGTCTCC-3′	5′-TCTGTTTCCACATCGTAGCG-3′
HMOX1	5′-CAACATCCAGCTCTTTGAGG-3′	5′-GGCAGAATCTTGCACTTTG-3′
GCLM	5′-CTCATTCCGCTGTCCAGGT-3′	5′-CCTTTGCAGATGTCTTTCCTGAA-3′
NQO1	5′-AGCCCAGATATTGTGGCCG-3′	5′-CCTTTCAGAATGGCTGGCAC-3′

**Table 2:** Differentially regulated oxidative stress-related genes in HUVECs following XXT treatment.

Gene	Description	GenBank accession number	Fold Change	*P* value
GCLM	Glutamate-cysteine ligase modifier subunit	NM_002061	6.25	0.0488
NQO1	NAD(P)H dehydrogenase, quinone 1	NM_000903	4.55	0.0004
TXN	Thioredoxin	NM_003329	3.70	0.0376
FTH1	Ferritin, heavy polypeptide 1	NM_002032	3.03	0.0081
GPX6	Glutathione peroxidase 6 (olfactory)	NM_182701	2.56	0.0449
HMOX1	Heme oxygenase (decycling) 1	NM_002133	2.50	0.0323
OXSR1	Oxidative stress responsive 1	NM_005109	2.17	0.0024
SELS	Selenoprotein S	NM_203472	2.13	0.0297

PTGS1	Prostaglandin-endoperoxide synthase 1	NM_000962	0.12	0.0227
DHCR24	24-Dehydrocholesterol reductase	NM_014762	0.21	0.0074
MB	Myoglobin	NM_005368	0.22	0.0390
CAT	Catalase	NM_001752	0.24	0.0021
UCP2	Uncoupling protein 2	NM_003355	0.25	0.0013
GTF2I	General transcription factor IIi	NM_001518	0.27	0.0013
PDLIM1	PDZ and LIM domain 1	NM_020992	0.30	0.0042
PRDX2	Peroxiredoxin 2	NM_005809	0.31	0.0422
GSTZ1	Glutathione transferase zeta 1	NM_001513	0.32	0.0014
